# The application of theories of the policy process to obesity prevention: a systematic review and meta-synthesis

**DOI:** 10.1186/s12889-016-3639-z

**Published:** 2016-10-13

**Authors:** Brydie Clarke, Boyd Swinburn, Gary Sacks

**Affiliations:** 1 Global Obesity Centre, Centre for Population Health Research, Deakin University, 221 Burwood Hwy, Burwood, VIC 3125 Australia; 2 Population Health & Prevention Strategy Unit, Prevention, Population, Primary and Community Health Branch, Department of Health and Human Services, 50 Lonsdale Street, Melbourne, Victoria 3000 Australia; 3 Population Nutrition and Global Health, University of Auckland, Victoria Street West, Auckland, 1142 New Zealand

**Keywords:** Obesity prevention, Policy, Food, Nutrition, Policy process

## Abstract

**Background:**

Theories of the policy process are recommended as tools to help explain both policy stasis and change.

**Methods:**

A systematic review of the application of such theoretical frameworks within the field of obesity prevention policy was conducted. A meta-synthesis was also undertaken to identify the key influences on policy decision-making.

**Results:**

The review identified 17 studies of obesity prevention policy underpinned by political science theories. The majority of included studies were conducted in the United States (US), with significant heterogeneity in terms of policy level (e.g., national, state) studied, areas of focus, and methodologies used. Many of the included studies were methodologically limited, in regard to rigour and trustworthiness. Prominent themes identified included the role of groups and networks, political institutions, and political system characteristics, issue framing, the use of evidence, personal values and beliefs, prevailing political ideology, and timing.

**Conclusions:**

The limited application of political science theories indicates a need for future theoretically based research into the complexity of policy-making and multiple influences on obesity prevention policy processes.

**Electronic supplementary material:**

The online version of this article (doi:10.1186/s12889-016-3639-z) contains supplementary material, which is available to authorized users.

## Background

The global obesity epidemic warrants urgent government action [1]. There is extensive literature advocating policy action for obesity prevention, with numerous studies outlining the various approaches available to governments [2–14]. Policy-led obesity prevention interventions are cited as having the potential to affect the whole population (including vulnerable or difficult to reach groups), as well as enabling desired changes to become systemic and, therefore, more likely to be sustained in the long term [2]. The World Health Organization (WHO) calls for policy action in the area of obesity, as articulated in documents such as the Global Action plan for the prevention and control of non-communicable diseases (NCDs) [15]. Accordingly, some countries have recently increased the use, and widened the scope of policy and legislative interventions to reduce obesity [16–19]. A recent review found 89 % of developed countries reported having a unit, branch or department in their Ministry of Health tasked with acting upon NCDs, including obesity [19]. High-level policy and strategy action has also been increasing with most countries now having a strategy or action plan on obesity or healthy eating [1]. Worldwide, policy action has included some ‘hard’ regulatory approaches such as mandatory standards for nutrition labelling, marketing restrictions, taxes on unhealthy foods, and financial incentives for production and retail of healthy food options [20]. However, ‘soft’ policy options have dominated; with a large number of countries implementing settings based health promotion programmes or social marketing strategies as their primary policy response to obesity [1, 20–22]. Yet despite numerous policy responses globally, no government has implemented a comprehensive set of policy approaches, which may explain, in part, the limited success in stemming the obesity epidemic [1, 12].

Policy change in support of obesity prevention faces political difficulty, particularly for ‘hard’ policy approaches [2, 23, 24]. There are numerous potential reasons for this difficulty, such as the power of food industry lobbying against the policies [25, 26], a perceived lack of evidence to support policy decision making [27], the lack of public pressure for policy change, and political ideology inhibiting the implementation of interventions deemed to infringe on the free market or personal liberties [25, 26, 28, 29]. As a result, obesity is often perceived to be policy resistant [30].

Nevertheless, there is limited evidence of the specific drivers of particular policy decisions. Accordingly, there have been calls to examine the processes surrounding the formulation and adoption of policy in support of obesity prevention [24, 31–35]. Recent studies conducted in Australia have explored the general barriers and enablers to implementing various policy approaches in support of obesity prevention [13, 32, 33, 36]. Similar studies have also been conducted in the US [37, 38], Fiji [39], and New Zealand [40]. These studies assist in identifying some of the policy process determinants that influence the policy decision-making processes related to obesity prevention. They include: individual skills, knowledge and capabilities of policy actors, and the processes within political institutions that shape policy adoption [13, 39]. Other factors identified as having an influence on policy decisions include the power dynamics of networks and groups involved in policy development, as well as socio-political and economic factors that shape individual policy maker’s ideas related to policy issues [32, 41]. These factors include shifts in macroeconomic conditions, mobilisation and strengthening of influential groups, and changes in the governing political party [42].

Whilst the aforementioned studies identify potential policy determinants, they do not provide a comprehensive understanding of *how* these determinants influence decision-making at various ‘stages’ of the policy process (i.e., agenda setting, policy formulation, implementation and evaluation [43]). For example, food industry power has been acknowledged as a critical factor, but most studies do not elucidate how the food industries’ influence is exerted and with whom. Furthermore, whilst many studies identify that media framing is important, they do not answer questions of how this influences the policy process. This reflects the need, recently identified by scholars, for political science theoretical perspectives to be used in order to better understand public policy decision-making related to obesity prevention [34, 44]. As Breton and de Leeuw (2011) suggest, “without proper theoretical grounding, successes and failure [in relation to altering policy] cannot be satisfactorily explained and remain all but just anecdotal accounts” [45] (p42).

Two recent reviews have investigated the use of theories to understand policy processes in this domain. Lyn and colleagues investigated ways in which policy science can inform obesity prevention change efforts [46]. However, they only looked at a limited number of policy science theories. Another recent review [47] examined the extent of use of political science theory to understand the influences on food and nutrition policy processes. This review found a limited number of studies that were underpinned by political science theory. However, no reviews have investigated the use of political science theories to understand obesity prevention policy processes more generally (e.g., including policies related to both nutrition and physical activity). Moreover, the previous reviews did not undertake critical appraisal of included studies, nor did the authors provide a meta-synthesis of the findings from included studies.

This study aimed to review the application of political science theoretical frameworks within the field of obesity prevention policy *and* synthesise qualitative findings in the area. In so doing, the study sought to answer the research question: what are the key influences on obesity prevention policy decision-making and how do they influence policy processes?

## Methods

### Study design

A two stage review strategy was adopted. Firstly, a narrative review of the political science literature was conducted to identify political science theories of the policy process.

Secondly, a systematic review of peer reviewed journal articles was undertaken, searching for studies that had applied theories of the policy process (as identified in the first stage of the review) to obesity prevention policy.

### Search strategy

#### Identification of theories of the policy process

For stage one, a search of relevant academic books and book chapters was conducted in order to develop a list of *theories of the policy process*. This was supplemented by a search within all issues of key policy journals (Health Policy and Planning, Health Policy, Journal of Health Politics and Law, Journal of Public Health Policy, Journal of Social Policy, Policy and Politics, Policy and Society, Policy Studies, Political Sciences, Public Administration Review, Social Policy and Administration and Milbank Quarterly) for the terms “theor* of the policy process” and “policy process theory”. Only articles published in English were reviewed. In order to be included in this study, theories of the policy process needed to: provide sufficient detail to enable application of the theory to an analysis of specific policy examples, be generalisable in nature (i.e., not for use within a specific policy area or country context only), and have been subjected to repeated evaluation (i.e., numerous applications of the theory published). See Additional file 1 for more information on identified theories. These elements were based on the criteria suggested by Sabatier for defining theories of the policy process [48]. This first stage of the literature search was undertaken by BC and was supported by a research librarian.

#### Application of theories of the policy process to obesity prevention

For stage two, BC conducted a systematic search of peer-reviewed literature was conducted using electronic databases, including PubMed, Academic Search Complete, CINAHL, SocINDEX and Health Policy Reference Centre (see Additional file 2 for complete list). Keywords that would retrieve obesity prevention policies included terms related to nutrition (e.g., food, nutrition, sugar, salt) and physical activity (e.g., leisure, sedentary). The MeSH database was used to identify other alternative terms. These were then combined with titles of theories of the policy process that were determined in the first stage of the review. The complete list of search terms can be found in Additional file 1. Reference lists of included studies were also reviewed to identify any additional studies, as well as a search for articles citing any included study.

### Inclusion of relevant studies

The titles and abstracts of all articles retrieved from the searches were screened. If there was insufficient information outlined in the title and abstract in order to make a decision regarding inclusion / exclusion then full texts were retrieved and reviewed against the inclusion criteria. Studies were selected if they:

1. Were empirical studies of policy processes in the government setting (all government levels and countries were accepted). It was acknowledged that this was likely to result in significant heterogeneity in studies but was deemed appropriate due to the exploratory and qualitative nature of the review.
2. Applied a theory of the policy process to understand factors that influence policy processes and decision-making. This application of theory needed to be explicitly stated, and could be related to the study design, data collection of data analysis phases.
3. Focused on policies related to the prevention of overweight or obesity or the proximal determinants of dietary and physical activity behaviours
4. Written in English
5. Published before July 2015.

Studies were excluded if they were:

1. Reviews or conference proceedings
2. Studies focusing only on policies related to the treatment of obesity (e.g., bariatric surgery).

### Quality assessment

All articles that met the inclusion criteria underwent a quality assessment, undertaken by BC. As the articles were all non-randomised qualitative studies, the Cochrane Collaboration’s guide for scientific rigour assessment in qualitative systematic reviews was deemed the most appropriate assessment tool [49].

This guide describes the need to assess:

- C*redibility* (truth value), which can be enhanced through the use of verbatim quotes, auditor or participant validation, or through the use of persistent observation.
- *Dependability*, which is enhanced through reporting details of data-sampling, -collection and -analysis that is logical and appropriate given the selected methodology. Furthermore, dependability is enhanced when the research is traceable by employed strategies such as peer review, debriefing, audit trails or triangulation.
- *Confirmability*, according to whether there was documentation of the researchers’ reflexivity to allow an assessment of the potential influence of their theoretical perspectives on the resulting presented findings; and
- *Transferability*, evaluating whether research findings are transferable to other specific settings through the provision of a ‘thick description’ of the research context.

However, the Cochrane guide does not prescribe checklist scores for each of the above constructs that could be used to provide an overall quality rating. In the absence of such a checklist, a rating of high (H), medium (M) or low (L) was assigned to each study for each of the above-mentioned criteria. This rating was based on the extent to which each study had demonstrated the quality strategies outlined for each criterion. See Additional file 3 for more detail on the scoring process for each quality criterion in order to allocate a high, medium or low score for each study. For example, in order for a study to be rated high for credibility, the study must have demonstrated the use of two or more of the following: verbatim quotes, auditor or participant validation or persistent observation. A low score was assigned when studies reported minimal or no application of the above quality strategies. A similar approach was used for the transferability, dependability and confirmability constructs.

### Data extraction

Data considered for extraction included all text labelled as 'results' or 'findings' in study reports [50]. However, for a number of studies there were no such headings provided [51, 52] or results were included in a “Discussion” section only [53, 54]. Therefore, the reviewer considered all data that appeared to be reporting results, and excluded text discussing the existing literature. Data was extracted in regard to the study year and setting, policy level/s and area, research design and methods including strategies used to increase rigor and trustworthiness (to enable critical appraisal to be undertaken). Other relevant strengths and limitations (e.g., whether ethics approval was obtained) were also extracted from each study. The ‘stages’ of the policy process for which the study focused, if specified, was also recorded. For example, whether there was a specific focus on agenda setting, implementation or evaluation. Whilst this is a simplification of the true complexity of policy decision-making, this was deemed useful when comparing study findings.

### Data synthesis

This review adopted a meta-synthesis approach whereby key ideas and concepts within each of the studies were identified [55]. This involved an iterative process of reading each of the studies and inductively coding findings, before re-reading, reflecting and grouping the elucidated findings into analytical themes [50]. The development of analytical themes focused on generating understandings or hypotheses that go beyond a description of the findings from included primary studies [50]. This stage of the meta-synthesis is shaped by the perspectives of the reviewer (BC) and is influenced by the research aims [50]. Therefore, it is important to note that this research adopts a constructionist epistemological position and an interpretivist theoretical perspective whereby learnings are informed by prior understandings and prejudices [56, 57].

BC conducted the search strategy, quality assessment, data extraction and synthesis. Peer debriefing with GS was conducted to support this process.

## Results

### Identification of theories of the policy process

In total there were 19 theories of the policy process identified. See Table 1 for a list of the theories and alternative names identified in the literature. Three of the theories identified (the Advocacy Coalition Framework (ACF), Multiple Streams Theory (MST) and the Punctuated Equilibrium Theory (PET)) have been described as ‘synthesis’ theories, in that they explicitly draw on multiple constructs from more than one other political science theories [58]. These ‘synthesis’ theories were often described in the literature as superior to other (non-synthesis) theories in providing an understanding of both policy stasis and change [48, 58, 59]. Reasons for this, cited in the literature, include their ability to better aid the understanding of complex decision making policy processes compared to traditional rational, linear models which depict discrete ‘stages’ of policy processes [48, 58–60], and their utility for conceptualising multifarious and interconnected concepts [48, 59].

**Table 1:** Theories of the policy process identified

Name of theory of the policy process	Alternative names identified in search
Advocacy Coalition Framework (ACF)	Advocacy Coalition Theory Sabatier
Multiple Streams Theory (MST)	Multiple Streams Framework Multiple Streams Analysis Three Streams Model Three Streams Framework Kingdon’s Theory
Punctuated Equilibrium Theory (PET)	Baumgartner and Jones'
Institutional Analysis and Development (IAD)	Ostrum
Institutional theory	Institutionalism Theory
Garbage Can Model	
Bacchi’s theory	What’s the problem represented to be? Bacchi’s approach
Agenda setting theory	
Incrementalism	
Rational Choice Theory (RCT)	Rational Choice
Actor Network Theory (ANT)	
Policy Network Theory (PNT)	
Theory of Collaborative Policy Networks	
Marxism	
Neo-liberalism	
Diffusion of Innovations (DOI)	
Narrative policy framework (NPF)	
Policy Feedback Theory (PFT)	
Social Construction Framework (SCF)	Social Construction Theory

A discussion each of the theories listed in Table 1 and their characteristics is beyond the scope of this paper (see John [58]; Sabatier and Weible [48]; and Cairney and Heikkila [61] for comprehensive reviews on the topic); however, a brief summary of each can be found in Additional file 1. Lumieux’s Theory of Coalition Structuring was identified in the review; however, it was excluded from this study as the details of the theory were not available in English.

### Application of theories of the policy process to obesity prevention

**Fig. 1:**
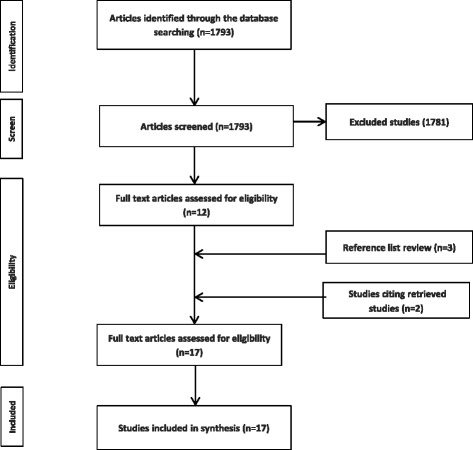
Systematic search strategy results

The systematic search identified 17 studies of obesity prevention policy that were underpinned by a theory of the policy process (refer to Table 2 and search process in Fig. 1). Of these studies, 13 studies applied a ‘synthesis’ theory, with 11 studies employing the MST theory and four the ACF. Two studies [52, 62] utilised multiple’synthesis’ theoretical perspectives, drawing upon *both* the ACF and MST. One study utilised the MST in addition to agenda setting theory. Four studies were underpinned by other theories of the policy process, drawing upon the Health Policy Analysis Triangle theory, the Diffusion of Innovations (DOI) theory, Institutional theory and the Narrative Policy Framework (NPF).

**Table 2:** Study characteristics and quality assessment of included studies

Author, year	Study setting	Policy level	Policy focus area	‘Stages’ of policy processes investigated *(agenda setting, policy formulation, implementation; evaluation; or any combinations of the above)*	Design and methods	Study participant information	Critical appraisal rating^a^				*Other strengths/ limitations (*e.g.*, sampling strategy; ethics approval)*
							Credibility *Do the findings represent the views of participant?*	Transferability *Were there contextual details provided?*	Dependability *Was the process logical, traceable?*	*Confirmability* *Are findings qualitatively confirmable through an analysis of audit trail?*	
Craig et al. 2010 [68]	Arkansas, United States of America (USA)	State level	Healthy eating environment policy Legislation to support healthy eating. The Act 1220 including the following components: • Child Health Advisory Committee (Education and Health representation). • Local regional schools PA and nutrition committee • State wide screening of BMI reporting back to parents • Vending machine legislation (restrictions) • Community health professionals within school setting	Stages of focus not specified however appears to focus on policy process stages leading to policy adoption (i.e., agenda setting and policy formulation)	Qualitative using secondary document data collected as part of a comprehend-sive evaluation of Arkansas Act 1220. Key informant interviews were also conducted with persons knowledge-eable of or involved in the passage of Act 1220.	No details were provided regarding study participants demo-graphics.	M	M	M-H	L	Secondary data source means that the theory did not inform the type of questions that were asked, potentially limiting what was able to be deduced. No details regarding ethics approval.
Dodson et al. 2009 [69]	Multiple states across USA	State level policy making (across several states)	General childhood obesity prevention legislation. Not a specific policy or set of policies.	Stages of focus not specified however appears to focus on policy process stages leading to policy adoption (i.e., agenda setting and policy formulation)	Qualitative study using interviews	There were 16 participants from 11 states, from various political parties, their professional background and length of tenure within their organisation, and geographical area represented also varied.	H	M-H	M	L	This was not a study of a specific policy process but rather of obesity prevention policy processes generally.
Freundenberg et al. 2015 [62]	Comparison of London and New York	Municipal level	Food policies, which included strategies to reduce obesity	Focused on election cycles providing opportunities for policies to be developed, and hence to stage of policy adoption only.	Document analysis	NA- document analysis	M	L-M	L	L	Secondary data source means that the theory did not inform the type of questions that were asked, potentially limiting what was able to be deduced.
Gladwin et al. 2008 [70]	Alberta, Canada	Provincial and local (local school board networks) and individual school level.	Daily physical activity mandatory requirements in schools as well as policy processes relating to decisions to not adopt the walking to school bus program.	Stages of focus not specified however appears to focus on policy process stages leading to policy adoption (i.e., agenda setting and policy formulation)	Qualitative comparative study of case study of two policies. Collected interviews (primary data) and documents related to the policy (secondary data).	None provided.	M	L-M	M	L	Only four of the interviewees were from the provincial level. The remainder were with parents, health professionals or school board members.
Gomez, 2015 [51]	Comparative study of USA and Brazil	National policy level	General obesity prevention policy.	Stages not specified however long term perspective allowed consideration of all aspects of the policy process (including feedback feeding into subsequent decision making)	Qualitative comparative case study drawing on secondary data sources of various documents (peer reviewed journal articles, government documents, and reports)	NA- document analysis	L	M	M	L	Secondary data source means that the theory did not inform the type of questions that were asked, potentially limiting what was able to be deduced.
Houlihan et al. 2006 [52]	England, and Wales, United Kingdom (UK)	National policy level	Policy focused on incorporation of physical activity/sport into school curriculum	Not specified however the use of two ‘synthesis’ theories could potentially include all ‘stages’	Qualitative study drawing on key informant interviews.	Nine participants in total, Including senior civil servants or senior members of interest/ professional organizations or senior academics.	M	M-H	L	L	No information regarding ethics approval.
Khayesi et al. 2011 [71]	Curitiba, Brazil	State level policy	Transport sector policy to increase active transport (through car dependence reduction policies)	No stages specified	Historical case study utilising documents. Two key informants assisted to inform the selection process of documents but did not provide any primary data.	NA- Document analysis	L-M.	M	L-M	L	Secondary data source means that the theory did not inform the type of questions that were asked, potentially limiting what was able to be deduced.
McBeth et al.2013 [72]	USA	Federal level policy	Obesity prevention policy generally	Agenda setting and the potential subsequent influence on policy formulation	Cross-sectional study documents (newspaper articles) using content analysis	NA- document analysis	H	H	H	L	Secondary data source means that the theory did not inform the type of questions that were asked, potentially limiting what was able to be deduced.
Milton et al. 2015 [75]	England, UK	National level policy	Walking promotion policy	Stages of focus not specified however appears to focus on policy process stages leading to policy adoption (i.e., agenda setting and policy formulation)	Qualitative case study drawing on document analysis and interview	Participants included representatives from relevant government departments and not for profit organisations, as well as, several independent consultants and other known advocates.	H	H	H	L	Details of ethics approval provided.
Mosier et al. 2013 [64]	USA, states of Colorado and Kansas	State level	Sales and excise tax policy on Sugar Sweetened Beverages (SSB)	Stages of focus not specified however appears to focus on policy process stages leading to policy adoption (i.e., agenda setting and policy formulation)	Qualitative comparative study, utilising observations, interviews and document analysis.	Nine individuals, involved in the policy processes were interviewed. No further details were reported.	M-H	M	M-H	L	No information regarding ethics approval.
Olstad, et al. 2015 [73]	Canada	State and provincial level	School based physical activity policy (legislation, rules, requirements)	All stages of policy process (including implementation)	Historical multiple case study. Systematic document review was used (no interviews or observation)	NA- no interviews	H	H	H	L	Secondary data source means that the theory did not inform the type of questions that were asked, potentially limiting what was able to be deduced.
Phillpots, 2012 [53]	England, United Kingdom	National policy	Sport and physical activity integration into school curriculum	All stages of policy process (including decision to cease the implemented policy)	Qualitative study design, drawing upon interviews, and document analysis.	Twenty-three interviewees from a range of government sport and education agencies who had been involved in the policy area for at least 5 years.	H	M	L-M	L	No information regarding ethics approval.
Quinn et al. 2015 [65]	King County, Washington, USA	Local level	Non-regulatory nutritional guidelines for food and beverages sold in vending machines.	Stages of focus not specified however appears to focus on policy process stages leading to policy adoption (i.e., agenda setting and policy formulation)	Qualitative case study design, using focus group, interview, and document review methods.	Focus groups: local health department staff interviews: Local Board of Health members, local elected, municipal staff, department directors officials, health expert from across 5 local jurisdictions	M	L	M-H	L	Ethics was obtained and details of the duration and timing of the interviews were given.
Reid and Thornburn 2011 [54]	Scotland, United Kingdom	National level	Physical education and activity policy	No stages specified, although clear focus on agenda setting	Field research involved key informant interviews	Participants from: various government departments (education, sport), local government sports development staff, relevant peak bodies, not for profit organizations, and politicians.	H	M-H	M-H	M-H	No information regarding ethics approval.
Thow et al. 2014 [63]	Ghana	National Level	A food standards policy to limit the amount of fat in meat and meat cuts	All stages from agenda setting, formulation, adoption and evaluation	Mixed methods case study	Participants were policy makers, implement-ers, producers, processors and retailers and respresented numerous government departments and stakeholder groups/ organisations	M	H	M	L	Ethics was obtained.
Ulmer et al. 2012 [74]	New Orleans, USA	State level	A Fresh Food Retailer policy Initiative	Stages of focus not specified however appears to focus on policy process stages leading to policy adoption (i.e., agenda setting and policy formulation)	Qualitative study using interviews	Participants were from various organizations and included city agency staff, city council members, grocers, representatives from trade associations and fınancial institutions, public health professionals, and food advocates.	L-M	L-M	L	L	No information regarding ethics approval.
Yeatman, 2003 [76]	Australia	Local level policies (four case studies)	Food policy	Stages of focus not specified however appears to focus on policy process stages leading to policy adoption (i.e., agenda setting and policy formulation)	Case studies using interviews and document analysis	Participants included local food policy councils, local elected members and local government middle managers.	L-M	M	L	L	No information regarding ethics approval.

^a^Score for each aspects found in rigorous and ‘trustworthy’ qualitative research [49]. ‘*L*’ indicates a low quality assessment, *M* medium, *H* high quality

### Study characteristics

#### Policy location and government level

The majority of studies were conducted in the United States (US) (*n* = 7), the United Kingdom (UK) (*n* = 4) and Canada (*n* = 2) (refer to Table 2). There was heterogeneity in regard to the level of government investigated, with several studies focused on national level policy (*n* = 7), and state/provincial level policy (*n* = 7), whilst two studies considered both local and provincial level policies, whilst one study focused on the local level only.

#### Policy area of focus

The most commonly studied obesity prevention policy areas were those that focused on the integration of physical education into schools (*n* = 5). There were also studies of overarching obesity prevention policy (*n* = 2), walking promotion/ active transport (*n* = 2), and food and nutrition/ healthy eating policies (*n* = 6), and single studies on vending machine policy, sugar sweetened beverage legislation and food standards to limit the amount of low quality meat in the food system.

#### Quality of studies

Only one study used a mixed methods case study approach to examine the entire policy process [63]. All other studies were qualitative in nature, drawing on documents (*n* = 11), interviews (*n* = 10), observations (*n* = 1) and focus groups (*n* = 1).

There was significant variation in the documentation of methods and often limited detail in regard to the strategies used to increase the rigour and trustworthiness of reported research. See Table 2 for assessment of each included study. Whilst many of the included studies increased *credibility* through the use of verbatim quotes from interview transcripts, no included studies reported utilising participant validation or persistent observation. Only a small number of studies provided detailed contextual information or participant demographics to enhance the *transferability* of findings. There was also substantial variability in the extent to which studies were *dependable*, with some studies triangulating data sources. Two of the included studies [64, 65] used three forms of data for triangulation, whilst six studies included two forms of data to inform findings. There was minimal evidence of peer review, dual coding, development of audit trails, or peer-debriefing. Few included studies provided enough detail to assess methodological and philosophical congruity. The *confirmability* of findings was also often limited in that there was minimal detail reported regarding researcher perspectives in all but one of the included studies. This impacts on the generation and interpretation of findings [66]. Only three of the 12 studies that used interviews as a primary data source reported details regarding human research ethics approval.

These methodological limitations are important to consider when interpreting the findings. It is acknowledged however, that the limited reporting of the above constructs is often due to space limitations set by peer reviewed journals, and the review process itself, rather than simply as a result of ‘poor’ research [67]. Given the limited evidence available with respect to obesity prevention policy processes, it was considered appropriate to synthesise the influences identified in the included studies despite their methodological limitations so as to provide a guide to areas of focus for future more rigorous qualitative research in this area.

#### Meta-themes from included studies

There were several themes that emerged from the included studies regarding the key influences on obesity prevention policy adoption (Table 3). Refer to Additional file 4 for more details of the findings of each of the included studies. These themes were: industry and stakeholder group and coalition influences; institutional factors, including feasibility of policy options; leadership of key individuals; narratives and framing; political ideology; personal values, beliefs and experiences; use of evidence; timing; and exogenous factors, such as crises and excessive budget deficits.

**Table 3:** Summary of themes of influence on obesity prevention policy processes, by included studies

Author, year	Theory used	Influences on policy processes								
		Coalition/ industry group lobbying	Political Institutions and political systems	Leadership of key individuals	Narrative and framing	Prevailing political ideology	Personal values & beliefs	Use of evidence	Timing	External socio-political (exogenous) factors
Craig et al. 2010 [68]	Multiple Streams theory (MST)	✓	✓	✓			✓	✓	✓	
Dodson et al. 2009 [69]	MST	✓	✓		✓	✓	✓			
Freunen-berg et al. 2015	MST and Advocacy Coalition Framework (ACF)	✓	✓		✓			✓		✓
Gladwin et al. 2008 [70]	MST	✓	✓		✓		✓	✓		
Gomez 2015 [51]	Institutional theory	✓	✓	✓		✓	✓	✓		
Houlihan et al. 2006 [52]	MST and ACF	✓	✓	✓	✓	✓		✓	✓	✓
Khayesi et al. 2011 [71]	MST	✓	✓	✓		✓	✓		✓	
McBeth et al. 2013 [72]	Narrative Policy Framework				✓					
Milton and Grix 2015 [75]	MST	✓	✓	✓	✓	✓	✓	✓	✓	✓
Mosier et al. 2013 [64]	MST	✓	✓	✓	✓	✓		✓		✓
Olstad et al. 2015 [73]	Diffusion of innovations theory	✓	✓	✓				✓		
Phillpots 2013 [53]	ACF	✓	✓	✓	✓	✓	✓		✓	✓
Quinn et al. 2015 [65]	MST	✓	✓	✓	✓	✓		✓	✓	
Reid and Thornburn 2011 [54]	MST	✓	✓		✓	✓	✓			
Thow et al. 2014 [63]	Health Policy Analysis Triangle	✓	✓		✓	✓		✓		✓
Ulmer et al. 2012 [74]	ACF	✓	✓	✓					✓	✓
Yeatman 2003 [76]	Agenda setting theory and MST		✓	✓				✓		✓

Authors identified that *coalitions*, *groups or networks* used various strategies which were key drivers of policy adoption. For example, established working groups, or committees embedded within political systems, were cited as influential in decision making processes, given their ability to shape proposed policy approaches [63, 68, 71]. In contrast, when policy groups or coalitions were not integrated within government decision-making structures, this was often identified as a limiting factor in their ability to influence the policy process [51, 54, 71]. Some studies identified that those groups on the periphery of policy-making processes attempted to influence policy through altering public awareness and shifting the dominant narratives regarding policy issues [51, 54, 71]. The effectiveness of such strategies in achieving impacts on policy adoption was seen as reliant on other factors such as direct lobbying to policy makers [68]. Lobbying was certainly identified as a successful mechanism for influencing obesity prevention policy in a number of the included studies [53, 64, 65, 75]. For example, Mosier’s study of sugar-sweetened-beverage tax policy found that lobbying was undertaken directly with policy decision makers, with appeals aligned to politicians’ ideological inclinations proving successful for policy change [64]. Industry group influencing of Ministers was also noted in a number of other studies [53, 65]. Lobbying directly to key leaders of relevant government departments was also identified as a useful strategy in the physical activity policy context [53].

Relevant groups and networks, seeking to influence policy, also jostled for greater power in decision-making circles than opposing or competing groups [53, 54]. However, these power struggles were often cited as detrimental to policy change being successfully implemented [53, 54]. For example, Reid and Thornton in their study of policies regarding physical activity in schools found that too many small groups with their own agendas meant that Ministers could easily ‘flick them away’ [54] (p309). In contrast, when similar, or even disparate and often competing groups or organisations were brought together, this proved beneficial for policy adoption [53, 62].

*Political institutional factors* were identified as critical influences, with organisational structures [76], interdepartmental collaboration [71, 76, 77] and policy feasibility [52, 68–71] identified in included studies. Feasibility was referred to in terms of whether there were clear accountability pathways and existing infrastructure available and engaged to support the implementation of the policy [52, 62, 68–72, 76]. Feasibility was also linked to the cost to implement [69], or likelihood of continued resource availability for implementation [70], and the probability of the policy being implemented within the political cycle [54]. Other institutional factors cited included policy capacity of key individuals [54], administrative turnover [54, 74] and overall governmental risk aversion [54, 68, 74].

Broader *political systems* characteristics that influenced policy adoption were the decision-making venue openness (i.e., opportunities for stakeholders to provide input and influence policy decisions) and the degree of decentralisation of public health policy processes [51].

The *leadership of key individuals* was another key theme of influence on obesity prevention policy identified in the included studies [51, 68]. Political champions who advocated for and led the development of proposed changes were cited as critical in a number of studies [52, 53, 65, 68, 73–75]. Such individuals were often Ministers or senior bureaucrats in positions of decision-making authority [53, 65, 68, 74]. Similarly, individual advocates who had “the ear of a Minister” were able to influence policy decision making [75] (p5). However, authors identified that key individuals required supportive contexts in order to facilitate policy action, particularly in respect to the other themes identified (e.g., supportive public narrative and institutional contexts, absence of group/network power struggles) [51, 52]. The motivations behind the actions of key individuals and leaders were linked to their experiences, values, beliefs, and political ideologies in the included studies [52, 65, 68].

The *narrative and framing* of particular policy options was identified as an influence on policy adoption in a number of studies. In particular, issue framing around personal choices and responsibility, and the relative importance of treatment compared with prevention responses, were both identified as a barrier to policy progress for obesity prevention [69, 70, 72]. Conversely, when leaders were able to shift the narrative to align with the dominant political ideology, or to promote issue clarity, progress was enabled [53, 54, 64, 70]. Two studies found that when the policy narrative was broadened to emphasise that the policy would achieve goals beyond the originally narrow issue focus, there was policy advancement [70, 75]. The role of the media in influencing the dominance of policy narratives was also identified as critical in a small number of studies [62, 64, 69].

*Political ideology* was demonstrated to influence policy decision making through both its influence on the acknowledgement of obesity as an issue (i.e., agenda setting) as well as the type of policy instruments adopted. Three studies identified that political ideology influenced the prioritisation of obesity prevention policy through historically held beliefs regarding the role of government in public health matters [51, 65, 75]. The degree of influence of Departments of Health relative to other government departments, such as Departments of Transport or Defence was also identified as important [52]. The type of policy instrument deemed acceptable for implementation was also identified as being influenced by political ideology [53, 54]. For example, neoliberal ideologies held by key policy actors provided barriers for the adoption of some proposed policies that were argued as potentially economically detrimental [53]. Similarly, the dominant ideological values of powerful groups were demonstrated to influence the progress of obesity prevention policy [51, 69].

*Timing* within broader political systems contexts was identified as a critical factor in some studies [53, 68, 74, 75]. Timing was also identified by authors as important in terms of the way in which a number of previously disparate factors can come together to facilitate policy change [52, 68]. For example, in their study of healthy eating legislation, Craig and colleagues reported that a change in the dominant beliefs of policy actors occurred prior to a key nutrition summit, which provided the decision makers with the opportunity to obtain timely relevant evidence of feasible policy options, as well as the required partnerships for implementation [68]. A number of authors identified that key individuals, coalitions and groups needed policy capacity to seize such policy opportunities, often doing so by pushing their preferred policy options as feasible and affordable strategies at critical times (e.g., at candidate elections) [52, 62, 70, 71]. This often required reframing the policy option to be more amenable to decision maker priorities at the time [52, 75].

*Use of ‘evidence’*, in its various forms and meanings, arose as an important influence on obesity prevention policy adoption, in a number of studies. Evidence of the need for action was one form of evidence cited as imperative [63, 70, 73]. For example, an increasing body of evidence regarding an issue was seen is important in pushing it higher on the political agenda [63, 73]. Evidence in terms of the effectiveness of alternative policy instruments or approaches was also seen as important [73]. Both forms of evidence were identified as tools used primarily to strengthen existing ideological arguments [52, 70]. Accordingly, one included study underscored that professional judgement and political ideology were stronger drivers of policy than evidence [75]. It therefore remained unclear, from the studies included, how, if at all, evidence of effectiveness of various obesity prevention policy options actually drives policy decision-making.

Finally, a number of studies suggested that *external factors* impacted policy actors’ ability to drive policy change. For example, studies found that time critical windows of opportunity were created by external influences such as budgetary crises [64] and extraordinary treasury reviews [75], natural disasters [74], and the Olympics being held [53, 75]. On the contrary, budget contractions were also identified as creating a barrier for implementation of new policy initiatives [53]. The influence of other government levels, such as national or state strategic directives dictating government priorities, was also identified as an external impact on policy decision making [76].

## Discussion

This review demonstrates that there has been limited application of political science theories of the policy process to the study of obesity prevention. The review revealed that, of the existing studies in this area, there was greater utilisation of the MST than other political science theories. Whilst some of the studies provided background to the theories employed and how this related to the research questions under investigation, none of the included studies reported the rationale for theory selection.

The review also revealed that, to date, most studies have been in the USA or UK context. Most studies did not delineate the stages of policy process under investigation, which is consistent with political science conceptualisation of policy as a complex, non-linear process [48, 58, 59, 79–81]. However; a number of studies did define the areas of focus, with some concentrating primarily on agenda setting only [54]. Such agenda setting focused approaches have been criticised for not adequately considering the complexity of how agendas, policy formulation and outputs are intertwined [58, 81]. For example, considerations of technically feasibility of policy instruments, which are normally undertaken following agenda setting phases, and which are demonstrated herein as an influencer of obesity prevention policy [52, 62, 68–70]) can also strongly shape decisions made by policymakers.

Studies included in this review had limitations, which impacted on the confirmability and dependability of their findings. These limitations included the minimal use of techniques such as participant validation, development of audit trails, peer de-briefing, provision of contextual information and data triangulation, all of which can be used to increase the trustworthiness of findings. Nevertheless, several themes related to key influences on obesity prevention policy were prominent across numerous studies, including the role of groups and networks; political institutions and political system characteristics; the framing of the issue; the use of evidence; personal values and beliefs; the prevailing political ideology; the timing of the policy process; political leadership; and external socio-political factors. These themes largely relate to constructs identified within the MST, which is at least partially because the majority of included studies employed this theory of the policy process and so analysed data in terms of MST constructs.

Whilst some of the influences identified (group and networks, external socio-political factors, and the role of key individuals) are congruent with those identified previously in a-theoretical studies of obesity prevention policy process [13, 31–33, 82], this review has illuminated additional effects on obesity prevention policy, such as personal values and beliefs and timing. Many of the studies included herein did not find strong evidence to support the notion that policy-maker knowledge or capacity to utilise evidence is a key influencer of obesity prevention policy adoption. This is in contrast to findings from some previous a-theoretical studies [33, 35, 38]. This indicates that, despite efforts from many in the public health community to increase evidence-based policy (EBP) [25, 35, 78], at least in the area of obesity prevention, the role of evidence during policy decision-making remains unclear, and other barriers and facilitators appear more important. Moreover, this review of theoretical studies begins to shed light on *how* such influences can both enable as well as prohibit, evidence informed obesity prevention policy progress.

It is also crucial to highlight that the key influences identified herein are not mutually exclusive and often interrelated. Hence, it would seem to be highly appropriate to use ‘synthesis’ theories of the policy process in future studies of obesity prevention policy. The application of these more comprehensive ‘synthesis’ theories of the policy process, within this context of obesity prevention, is broadly consistent with the recent political science literature, including the findings of a previous review focused on nutrition policy [47], where policy scholars have noted that synthesis theories are superior in explaining how and why policy stasis or change occurs [48, 58, 59]. The ACF, MST and PET, as synthesis theories, consider numerous important influences on decision-making, including the role of decision making rationality, ideas, institutional aspects, groups and network influences, as well as external socio-political factors. They also draw attention to how these influences are interconnected to better explain the potential mechanisms for policy adoption [83].

The large number of studies utilising the MST is consistent with the broader political science literature where there has been extensive application of this theoretical approach [84]. The MST has been commended as a comprehensive theory for its consideration of the role of ideas, institutions, exogenous factors and decision maker rationality [58, 61, 85]. However, the MST has also received criticisms in regard to its limited exploration of group and network power dynamics [58], which were often identified, in included studies, as influential aspects of the policy process. Therefore, it is plausible that the use of MST in such studies may have resulted in the important findings related to structuring and power dynamics of groups and networks being underexplored to some extent [58]. Furthermore, the MST has been criticized for paying insufficient attention to the overall political climate [86–88], which has been identified in the obesity prevention literature as potentially impacting policy progress [28].

The small number of studies applying the ACF to this context of obesity prevention is somewhat surprising, given the ACF’s value in exploring the role of group power dynamics in policy processes [89]. Furthermore, this framework has had widespread application across various policy contexts, including health, and is perhaps the most empirically tested theory of policy process [89, 90]. Indeed, the ACF has been identified as a robust theory of the policy process, and is praised for its consideration of numerous constructs including ideas, institutions, groups and networks, as well as, external factors [58, 61, 89, 91]. However, the ACF has also been criticised, with policy scholars highlighting the limited focus on the role of individuals and institutions within the ACF. [61, 92] Whilst the most recent iterations of the framework suggest that institutional factors are integrated [86, 89], it is argued that these remain focused only on intergovernmental relations rather than considering how political system structures and norms can influence individuals [58]. Both of these factors have been identified previously as important influences of obesity prevention policy [13, 39].

With theories of the policy process continuing to emerge and develop over time, the findings from original empirical applications of a theory may differ if they were to be repeated with the incorporation of constructs that were added to the theory at a later stage [48]. Hence, by applying refined theories, further knowledge can be created to support future policy development [48].

Given the noted limitations of ‘synthesis’ theories [58, 93], policies scholars have suggested in addition to utilising refined theories of the policy process, using multiple perspectives can provide a more comprehensive understanding of the complexities involved in policy process [90, 93, 94]. Two included studies [52, 62] employ this approach, utilising both the MST and ACF theories.

There were four studies included in this review that did not draw on synthesis theories. Two of these [72, 73] focused primarily on the role of ideas through their utilisation of the NPF and the DOI. Whilst the role of ideas in obesity prevention policy processes has been identified as integral in the obesity prevention literature [95–98], such theories have been criticised given their limited consideration of institutional and power influences of policy [48, 58]. For example, the NPF, with its focus on the role of ideas and how these affect agenda setting, may illuminate the dominant narratives surrounding a policy issue, however it arguably falls short in demonstrating the impact of such narratives on decision maker behaviour [58, 99]. Policy decisions can be informed by narratives, *in addition* to other issues of practicality linked to institutional processes and competing government policy priorities [58]. In contrast, whilst Gomez’s [51] study, that employs Institutional theory (another non-synthesis theory), does address these institutional influences on policy decisions, it can also be criticised given it largely under-theorises the role of ideas, or groups and networks and their exercising of power to influence policy decision makers. The final of these four studies, by Thow and colleagues [63] was underpinned by the Health Policy Analysis Triangle. While the simplicity of this framework may be useful, it may also result in the inadequate illumination of the complex interrelationships between various policy influences [100].

This review was strengthened by the systematic approach to searching for studies that had applied theories of the policy process to the area of obesity prevention. This search was aided by a research librarian and was conducted across a large number of databases. An audit trail of the literature search, data extraction and meta-synthesis findings have been provided (see Table 1 and Additional files 2 and 4) to enhance the credibility of the study [49]. In addition, the use of peer debriefing, researcher reflexivity and the declaration of the analysing researcher’s epistemological position helps to increase the dependability and confirmability of the findings [49].

However, there are a number of limitations. Firstly, the search for theories of the policy may not have been exhaustive. Nevertheless, it was arguably more extensive than previously conducted reviews in this area. [46, 47] Secondly, the systematic search may not have been completely comprehensive due to inadequate indexing of terms for qualitative research in bibliographic databases, which can result in incomplete search results [101]. This has also been a limitation acknowledged previously within the context of public health systematic reviews [101]. In order to mitigate this limitation, we conducted the systematic search for all years and for all text (in some cases) rather than titles or abstracts only, as per recommendations from the Cochrane Collaboration for searches of this nature [101].

As noted earlier, the critical appraisal, data extraction and data analysis was undertaken by one member of the research team (BC). The critical appraisal may have been strengthened two members of the research team undertaking this process, however time and resources precluded dual assessment. Similarly, dual coding, during the data extraction and analysis, phases may have enhanced the trustworthiness of the research, however the necessity for, or appropriateness of, this strategy within the qualitative research paradigm remains contentious [57].

The review is also limited by the paucity of evidence available in this area, which meant that stringent quality criteria were not used to exclude weak studies. The methodological quality was reported (refer to Table 2) to allow the reader to assess potential weaknesses in study results. Secondly, the breadth of studies, from multiple settings and contexts, presented challenges in terms of contradictory epistemologies, ontologies or methodologies [55]. The varied reporting of theoretical perspectives in the included studies made it difficult to ascertain the epistemological variability; however, it is likely that substantial heterogeneity was present. While the heterogeneity may have influenced the reliability of the meta-synthesis, the approach taken in this paper is supported by other authors, who suggest that combining findings from various epistemological approaches can increase the truth-value of meta-synthesis [102].

There was also heterogeneity in the different policy instruments and policy areas investigated in the included studies. There is likely to be some underlying differences in the policy influences in these different areas, and hence the key influences on policy adoption may also vary by area [45]. For example, whilst the role of group and network influences on decision making was identified as a key policy driver in most of the included studies, irrespective of policy focus, the ways in which group and networks structuring and power played out in food and nutrition focused policies may be substantially different to how they play out with respect to physical activity policies. It is therefore recommended that future robust and theoretically based research be conducted across numerous types of obesity prevention policies, in respect to both physical activity and healthy eating.

This review highlights the value of bringing a political science approach to the study of obesity prevention policy to help inform practitioners and policy makers regarding how policy decision making occurs in this area [103]. The study builds on previous a-theoretical research on obesity prevention policy to provide an overview of the current literature, which has utilised political science theory to better understand policy stasis or change. Without application of such theories, studies may be limited in their explanatory value of why certain policies responses to obesity are adopted whilst others are not [44, 104]. In contrast, the findings from theoretical studies, such as those presented in this review, may be better able to inform actors attempting to influence future obesity prevention policy as to the potential policy leverage points, as well as *how* to act to influence policy decision making [44, 104]. Given the direct policy relevance of such evidence, future high quality research in this area is particularly warranted.

## Conclusions

This systematic review investigated the application of political science theories of the policy process to the study of obesity prevention policy. The study found a number of political science theories available; however, there has been limited application of these theories within this policy area. Where political science theories have been applied to understand policy processes with respect to obesity prevention, the studies had substantial methodological weaknesses, particularly in regard to credibility and dependability of findings. Nevertheless, the meta-synthesis identified a number of key influences on obesity prevention policy decision-making that can be used to guide future investigations. The review highlighted the complexity of decision-making in relation to obesity prevention policy, and therefore recommends that future rigorous empirical investigations incorporate multiple theoretical perspectives to better guide policymakers as to potential leverage points and effective ways to influence obesity prevention policy.

## Additional files

Additional file 1: Theories of policy process. (DOCX 79.8 kb)
Additional file 2: Search strategy. (DOCX 18.2 kb)
Additional file 3: Critical appraisal rating system. (DOCX 19.3 kb)
Additional file 4: Detailed findings by study. (DOCX 50.9 kb)

## Abbreviations

ACF: Advocacy Coalition Framework
ANT: Actor Network Theory
DOI: Diffusion of Innovations
IAD: Institutional Analysis and Development
MST: Multiple Streams Theory
NPF: Narrative policy framework
PET: Punctuated Equilibrium Theory
PFT: Policy Feedback Theory
PNT: Policy Network Theory
RCT: Rational Choice Theory
SCF: Social Construction Framework
