# Supplementary material for: The application of theories of the policy process to obesity prevention: a systematic review and meta-synthesis
Source: BMC Public Health. 2016 Oct 13;16:1084. doi: 10.1186/s12889-016-3639-z (PMC5064928; doi:10.1186/s12889-016-3639-z)
Supplement: Additional file 1: — Theories of policy process. (DOCX 79.8 kb) [file 12889_2016_3639_MOESM1_ESM.docx]

| **Theory of the policy process** | **Key elements** | **Strengths and weaknesses** |
| --- | --- | --- |
| Actor Network Theory (ANT) | Is a comprehensive model of the innovation process which aims to “follow the connections between all actors (human and non-human) involved in a given situation and to trace the circulation of the ideas and interests of these actors and the transformations of the network” (1, p. 85). The framework which is based on the premise of actor networks (different to social networks) is useful for investigating intersectorial initiatives. The theory considers ‘problematisation’ of issues whereby the primary actor(s) define their problem, objectives, and fellow entities in a way which portrays their common goal as the necessary solution (1). Hence issue framing is central to the framework. Public opinion also feeds into this process (2). | The ANT was not developed for understanding public policy and as a result the theory has been criticised for its ability to adequately considering power relations that effect policy decision making (1). Therefore the frameworks is limited in that it does not account for how individuals have capacity to take action given variations in structures and power in policy processes, and how these evolve over time (1). |
| Advocacy Coalition Framework (ACF) | Sabatier developed the Advocacy Coalition Framework (ACF) to explain the emergence of changes in public policy (3). The ACF suggests that public policy alterations are driven by coalitions, of individual policy actors, including legislators, civil servants, journalists, academics, members of not for profit organisations and members of the courts (Jenkins-Smith et al., 2014). The theory assumes that policy actors are ‘boundedly rational’ and, due to limited cognitive abilities, they simplify the world through their existing belief systems to potentially bias the interpretation of information, evidence and experience (4). The ACF suggests that coalitions form based on common beliefs, and that they aim to disseminate information and influence policy in line with their key beliefs (John, 2012).  The framework postulates that external ‘shocks’ can give rise to changes in formation, and/or the balance of power of networks and coalition structures so that a new goal or a course of policy action is enabled (5, 6). However, coalitions with inadequate skills for action can fail to harness external system changes to drive major policy change (7). Furthermore, not all policy change is radical, hence the framework recognises that policy change can occur as a result of policy learning (3), whereby the beliefs of policy actors can be altered with experience and/or access to new information (8, 9). | A criticism of the ACF is the limited extent to which the framework addresses the role of individuals and institutions. Whilst later iterations of the framework (4) suggest that institutional factors are integrated, it is argued that these remain focused only on intergovernmental relations rather than considering how political system structures and norms can influence individuals (10). Largely the application of the ACF has been of policy processes over long time periods of time (10 years minimum is recommended) however this should not preclude studies of shorter duration (4).  Furthermore, Breton et al (11) suggest that the ACF is insufficient for answering questions such as “how did the advocacy coalition take advantage of opportunities and convert them into real gains?” or “How did they ensure that the policy subsystem adopted the core elements of their vision”. They suggest that the Lemieux’s theory of coalition structuring is better for answering such questions (11). However this has not been made available in English. |
| Agenda setting theory | A broad group of theories (for example both the PET and the MST have been referred to as applications of agenda setting theory) that focus on the role of active policy participants, as well as the processes through which issues rise to prominence as problems requiring government intervention (12) | Given its focus only on agenda setting, these models have received criticisms in regard to completeness in understanding policy change (10). For, example it may be that the rising of a policy issue on the political agenda couples with a window of opportunity that ultimately allows for policy change to occur. Without looking at issues of timing or institutional processes facilitate policy change to occur such models may not enable policy processes to be completely understood(10). |
| Diffusion of Innovations (DOI) | This theory considers how policies diffuse across states and other jurisdictions. Hence it focuses on the role of ideas within the policy process The focus of the theory is on discrete policy systems, containing states that may influence the propensity of other states adopting policies (13). The DOI outlines that diffusion occurs through: learning, imitation, normative pressure, competition, and coercion (13). These can occur in isolation or in combination (13). | The DOI has most frequently used at the USA federal therefore it is unclear how useful the framework is for state and local level policy decision making. It has been suggested that the role of groups and networks is not adequately explored and hence the framework is strengthened through the use of additional theories to better explain policy process (14). |
| Garbage Can Model (GCM) | The GCM (GCM) theory rejects rational model and incremental approaches and instead suggests that policy decision is ad hoc and haphazard. The model recognises that goals are often unknown by decision makers as is the causality of policy problems (15). The theory also suggests that what policy change occurs at any given point in time is the result of a fortuitous timing, with agendas and policy solutions all playing an important role  Also of note is the fact that the GCM heavily informed the MST (10). | The GCM has been applied primarily to US decision-making. Whilst some policy decision making is as “random” and irrational as those described by Cohen, March and Olsen, there are other times where policy making is more structured and ordered, and it is argued that the framework does not adequately allow for understanding decision-making in this context (16). The more comprehensive MST is therefore a preferred theoretical framework to the GCM (10). |
| Health Policy Analysis Triangle | This framework is a highly simplified approach to a complex set of inter-relationships influencing policy. The health policy analysis triangle focuses on actors, context and the content of policies (17). The actors refer to individuals or members of groups or organizations within policy communities. These actors differ according to power structures and their values(17). The context relates to many factors such as instability or ideology, by history and culture with the context influencing how issues arise on the agenda. This political science framework also seeks to examine the policy content (17). | Whilst this simplistic model provides useful insight into policy making processes the lack of detail, particularly regarding the interrelationships of the frameworks components(17), can be a limitation in explaining policy decision-making. |
| Institutional Analysis and Development (IAD) | The IAD considers how individual policy actors make decisions within institutions which affect human behaviour (18). The focus unit of analysis for the IAD is the “action area” which is determined by the analyst, as per the context (19). The framework can be useful for examining inter-organisational arrangements and relationships that influence policy adoption (19). The framework considers a number of criteria including efficiency, equity and accountability, as well as, focusing on the operational rules that impact upon decision makers (14). The IAD also considers the broader context, in regard to how social norms and shared preferences influence the rules, operational norms and individuals (14). | The role of networks and group structuring is implicit rather than explicitly outlined within the framework (14). The theory has also been criticised in regard to the limited guidance available for its application, and ambiguity in relation to definitions for the frameworks constructs and how to measure or operationalise these (19). |
| Incrementalism | Incrementalism theory is useful in that it guides examination of the decision making processes, as proposed by political parties, discussions in legislatures and those implemented by bureaucracies(10). Incrementalism suggests that policy results from the bargaining between stakeholder groups (20). The theories developer Lindblom suggests that citizens have minimal role in policy development and that interest groups often cancel out each other’s influence (21). | Incrementalism has been denounced for its inability to recognise power struggles between groups aiming to influence policy processes (10). It has also been critiqued for not recognising radical policy change which can be the result of external influences such as changes in ideas, economics and variations in the influence of interests (10, 22). |
| Institutional theories/ New institutionalism | Theories that focus on institutionalism, see political organisations, such as parliaments, and bureaucracies as crucial to the construction of public decisions and outcomes (16, 23). They also acknowledges that other aspects such as electoral systems and the internal rules of legislatures as fundamental in policy processes (10). Furthermore these theories suggest that institutional cultural norms and values also shape decisions (24, 25). As such, institutional theories, acknowledge that norms shape the “appropriateness” of policy actions within predefined categories (26). Ultimately institutions “constrain and corrupt” human decision making as well as inducing behaviour (23, p.9). Institutional theory or new institutionalism analysis therefore focuses on how preferences and decisions are artefacts of institutions (23, 27). | Institutional theories have been criticised for being overly descriptive in terms of administrative details and formal procedures. (10). Furthermore, the political power studied, through the use of institutional frameworks, is limited to the institutional setting and does not consider partisan or industry in discussions that influence policy (23). |
| Marxism and Neo-liberalism | Also not explicitly theories of the policy process, Marxism and Neo-liberalism, have been used in policy studies to explore the exogenous impact of economy on politics. Particularly they help to explain how the interests of the private owners of capital, influence the practice of politics, suggesting that “broad changes in the structure of the economy are responsible for shifts in policy and in the framework of exercising power”(10, p. 88). Marxism and Neo-liberalism conceptualisations consider the role of private industry in shaping policy through the capitalist class and networks of supporting elites (10). Therefore, these perspectives can provide insights into the preferences that come to be expressed in politics and the outcomes that result from these political processes (i.e. class origins of elected officials or class biases in their decisions) (23). | These theories are most suited to comparative work, or work undertaken on a global level (10). They do not explain the complexity and variation of public policy, largely due to the limited consideration of the institutional factors and therefore they have been critiqued for not being suitable for accounting for the difference between sectors (10). |
| Multiple Streams Theory (MST) | This theory suggests that policy formation is the result of three sets of processes: problems, policies and politics. The problem stream, relates to issues that require action (28), as determined by numerous factors including availability of indicators and information, how issues are framed, current government conditions (e.g., budget deficit versus surplus) and prevailing political ideology (29). Therefore, values and beliefs are central to the MST (29). The MST suggests that problems can reach the attention of policymakers through focusing events (e.g., crises or changes of government) and feedback (e.g., policy diffusion across regions or countries) (29). The theory also suggests that policies are shaped by ideas generated by those who have an interest in the problem, such as academics, bureaucrats, politicians and interest groups (29). Some policy ideas can make it to the decision making stage unaltered; however, most are shaped or absorbed into other policy responses, or disappear completely (29).  The policy stream focuses on the solutions available to address identified problems, which take time to develop and evolve as numerous policy actors shape them. The third stream refers to the broader political discourse. This is influenced by national mood, pressure group advocacy, and administrative or legislative turnover(30). Changes in the politics stream are seen as the most powerful for reshaping the policy agenda (29).  The MST is premised on bounded rationality of policy actors, however the framework also acknowledges that policy systems contain numerous subsystems which facilitate concurrent “parallel processing” of various issues (28). As many issues vie for attention of senior decision makers, ‘policy entrepreneurs’ need to act swiftly when a ‘policy window’ opens in order to get their policy proposals to decision makers, or generate attention to their policy problem (7, 28). | The MST has been criticised for its limited application outside of the American political context or beyond national agenda setting processes (pre decision processes rather than all policy processes). The MST has also been criticised for paying little attention to group and network collaborative efforts working towards a specific goal and how this influences policy change (10), nor the broader political climate (31-33). |
| Narrative policy framework (NPF) | The NPF looks at how narratives influence public opinion, how these narratives are structured and how they reflect policy beliefs (14, 34). The role of political ideology can therefore be illuminated through NPF studies (34).  The NPF can be used to explore how narratives are used by actors (interest groups and elites) to expand their power to ultimately determine policy decisions (34). | The NPF has also been criticised for not adequately considering the role of institutions of policy settings. Furthermore, the micro-setting of the NPF limits the frameworks ability to determine *how* narratives influence the public policy process (34). Hence, it is argued that a complete understanding of policy process can only be obtained when the NPF is combined with other theories (14). |
| Policy Feedback Theory (PFT) | The PFT examines policy formulation and change, with a focus on policy design and dynamics such as feedback of policies into society (35). It suggests that policy commitments made in the past produce increasing returns and therefore make it more costly for different policy paths to be taken (14). The role of groups and networks structuring and power is central as is the role of ideas, with the framework suggesting that policy frames or institutionalised beliefs regarding a policy issue are key influences of policy adoption (14, 36). Much of the PFT applications have focused on the mechanisms in which implemented policies influence subsequent political discussions (36). | Whilst the PFT has a broad focus on ideas, networks and actors (14), the theory has received critiques. These have mainly being in relation to methodological issues, and the limited application to date (36). For example, there remain queries regarding selection bias of groups/ organisations who adopt policy approaches, which it is argued that this can influence how subsequent feedback and future policy adoption occurs (36). |
| Policy Network theories | Policy network theories suggest that organisations exchange resources and depend on each other in policy processes, which influences how policies are made and implemented (10). Policy network theorists have described issue networks which are open and dynamic, in terms of organisations involved (37), whereas policy communities are more stable and restricted networks that are responsible in delivering policy outputs (10). It is these aspects which are the focus of policy network investigations. | Policy networks theories have been criticised in their ability to *explain* policy change, given this may not be due to the role of the networks necessarily, but rather the influence of ideas, interests (and particularly the role of power in relation to this) or institutional factors (10, 38). Hence, such theories have been referred to as descriptive only (10). |
| Punctuated Equilibrium Theory (PET) | The PET is used to understand how incremental policy change is occasionally disrupted by exogenous factors such as shifts in dominant societal values and macro political context (25). The framework, like the MST and ACF, is premised on the notion of bounded rationality of human decision-making. It contends that, although policy subsystems enable parallel processing, high profile policy issues must be handled through serial processing (39). The PET suggests that parallel processing in numerous policy subsystems leads only to incremental budgetary changes (39). Therefore, access to high levels of decision making (e.g., national level politicians) is a prerequisite for significant policy change (39).  The role of institutions as fundamental in influencing policy, suggesting that ‘policy monopolies’ exist due to institutional structures whereby discrete policy teams are responsible for specific issues (39). It is postulated that through negative feedback processes, institutional policy monopolies are resistant to change, and hence the policy status quo is maintained (39). Therefore, the PET framework focuses on the conditions that lead to sufficient pressures being applied to political systems that result in either redefinition of the issue, or changes in the constituents or power balance within policy systems, to enable significant shifts in policy (39). The PET suggests that ‘policy images’, that is, the information driven and emotive appeals surrounding a policy issue, must be elevated to the macro political setting in order for significant change to occur (39). | The PET does little to explain the processes surrounding *how* these changes occur (25). Furthermore, Baumgartner and colleagues argue that the theory has most utility when applied to comparative studies, or those which compare policy systems characteristics (39). This is a result of its development, in which it was used primarily to explain governmental budgets (10, 25). Although the framework does refer to aspects such as the political agenda, and competing values, there is not a strong sense of *how* these influence policy outcomes (25). |
| Rational Choice Theory (RCT) | RCT assumes that individuals are utility maximisers and therefore decisions will be made in clear and consistent ways (29). As such, the RCT assumes that human beings preferences are fixed (at least for periods of time) and that individuals act to advance their welfare (e.g. through increasing their income) by balancing out current and future factors (10, 40). | It has been extensively argued that the assumption that individuals have the capacity to act as rational agents, is an oversimplified model that does not take into consideration the influences of other people and groups which aim to influence the policy process (10, 41). Similarly, it has been criticised for not taking into account organisational and bureaucratic limitations that influence decision making(10). |
| Social Construction Framework (SCF) | The SCF (like the NPF) recognises that humans draw from emotions when making decisions (14). The framework focuses on framing and assigning values and the emotive appeals regarding problems, and how this influences policy, particularly through agenda setting (42). The SCF also suggests that policy makers use evidence to support such emotional depictions (14). It suggests, when no strong social constructions are apparent, the influence of science is likely to be greater (43). The political hegemony that is shaped by the media is therefore central to the SCF (14). The framework also suggests that policymakers views of groups over time, contributes to their relative power held, which may remain consistent over a number of years without external shocks (such as economic crisis) (14, 42). | The SCF has also been criticised for not adequately exploring the role of institutions (44).  Furthermore, the theory has been criticised in that it may fail short in providing *causal* explanations of how ‘target’ populations are socially created within the policy context(44). This shortcoming has implications for understanding how to intervene in future policy change efforts (44). |
| Theory of Collaborative Policy Networks | This theory examines the compositions of stakeholder groups, the partnerships between stakeholders, as well as, the degrees to which these stakeholders and networks are embedded within institutionalised structures and how this influences policy development, adoption and implementation (45). The theory also looks at the characteristics (e.g. degree of collaborative leadership) of the policy networks that influence this process (45). | Given its focus purely on the nature of networks in influencing policy, other factors such as political ideology and ideas appear under-theorised in this framework. |
| What’s the problem represented to be? (WPR) or Bacchi’s Theory | The WPR framework considers how a problem is “problematized” and how this influences discourses surroundings the issue and ultimately policy decision making (46).The framework focuses on discourse and power predominately and comprises the following questions (47, 48): 1: What is the problem represented to be in a specific policy proposal and/or in policy debate? 2: What presuppositions or assumptions underpin this representation of the problem? 3: What effects are produced by this representation of the problem? What is likely to change with this representation of the ‘problem’? What is likely to stay the same? Who is likely to benefit from this representation of the ‘problem’? 4: What is left unproblematic in this problem representation? Where are the silences? How would ‘responses’ differ if the ‘problem’ were thought about or represented differently? (Here it is useful to think about shifts in representation of ‘the problem’ over time or across cultures); 5: How/where are dominant problem representations disseminated and defended? How could they be challenged? | Clearly delineated propositions that allow for testing and application. The focus on narrative discourse has resulted in criticisms of the framework in terms of not necessarily providing understanding of *how* discourse/ideas surrounding a policy ‘problem’ go beyond just being argued for (by various policy stakeholders) and actually reach policy adoption (10). In other words, this framework arguably does not provide enough guidance as the impact of ideas on policy decision making (10). |

**References**

1. Potvin L, Clavier C. Actor-network theory: the governance of intersectorial initiatives In: Clavier C, de Leeuw E, editors. Health Promotion and the Policy Process. Melbourne Oxford University Press 2013. p. 83-104.

2. Young D, Borland R, Coghill K. Changing the Tobacco Use Management System: Blending Systems Thinking with Actor-Network Theory. Review of Policy Research. 2012;29(2):251-79.

3. Weible CM, Sabatier PA, McQueen K. Themes and Variations: Taking Stock of the Advocacy Coalition Framework. Policy Stud J. 2009;37(1):121-40.

4. Jenkins-Smith H, Nohrstedt D, Weible CM, Sabatier PA. The advocacy coalition framework: foundations, evolution and ongoing research In: Sabatier PA, Weible CM, editors. Theories of the Policy Process: Westview Press 2014.

5. Sabatier PA, Weible CM. The Advocacy Coalition Framework: Innovations and Clarifications. In: Sabatier P, editor. Theories of the Policy Process. 2nd ed. Boulder Westview Press; 2007.

6. Cairney P. How can policy theory have an impact on policy making? The role of theory-led academic–practitioner discussions. Teach Public Adm. 2014.

7. Ameringer CF. Federal Antitrust Policy and Physician Discontent: Defining Moments in the Struggle for Congressional Relief. J Health Polit Policy Law. 2002;27(4):543.

8. Sabatier P, Weible CM. Theories of the policy process Oxford: Westview press; 2014.

9. Gagnon F, Turgeon J, Dallaire C. Healthy public policy A conceptual cognitive framework. Health Policy. 2007;81(1):42-55.

10. John P. Analysing public policy New York Routledge; 2012.

11. Breton E, Lucie R, Gagnon F, Jacques M, Bergeron P. Coalition advocacy action and research for policy development In: Clavier C, De Leeuw E, editors. Health Promotion and the Policy Process. Oxford Oxford University Press 2013. p. 44-63.

12. Eissler R, Russell A, Jones BD. New Avenues for the Study of Agenda Setting. Policy Stud J. 2014;42:S71-S86.

13. Rogers EM. Diffusions of innovations. 5th edition ed. New York: Free Press 2003.

14. Cairney P, Heikkila T. A comparison of theories of the policy process Oxford: Westview Press 2014.

15. Cohen MD, March JG, Olsen JP. A Garbage Can Model of Organizational Choice. Administrative Science Quarterly. 1972;17(1):1-25.

16. Howlett M, Ramesh M, Perl A. Studying Public policy: Policy cycles and policy subsystems. New York Oxford University Press; 2009.

17. Buse K, Mays N, Walt G. Making Health Policy London: McGraw-Hill Education; 2005.

18. Ostrom E. Background on the Institutional Analysis and Development Framework. Policy Stud J. 2011;39(1):7-27.

19. Ostrom E, Cox M, Schagler E. An assessment fo the Institutional Analysis and Development Framework and introduction of the Socio-ecological systems framework In: Sabatier P, editor. Theories of the Policy Process. Boulder Westview Press 2014.

20. Richardson J, Jordan A. Governing under pressure: the policy process in a post-parliamentary democracy. Oxford Oxford Publishing; 1979.

21. Lindblom CE. Politics and Markets New York Basic Books 1977.

22. Dror Y. Muddling Through-"Science" or Inertia? Public Adm Rev. 1964;24(3):153-7.

23. Immergut EM. The Theoretical Core of the New Institutionalism. Politics & Society. 1998;26(1):5-34.

24. Smith KD. Beyond evidence based policy in public health: the interplay of ideas Melbourne: Palgrave Macmillan 2013.

25. Hassenteufel P, Smyrl M, Genieys W, Moreno-Fuentes FJ. Programmatic actors and the transformation of European health care States. J Health Polit Policy Law. 2010;35(4):517-38.

26. March JG, Olsen JP. Elaborating the “New institutionalism”. In: Rhodes RAW, Binder SA, Rockman BA, editors. The Oxford Handbook of Political Institutions Oxford: Oxford University Press; 2006. p. 3-20.

27. Béland D. Ideas and Social Policy: An Institutionalist Perspective. Social Policy & Administration. 2005;39(1):1-18.

28. Zahariadis N, editor. The Multiple Streams, Framework- Structure, Limitations, Prospects Boulder: Westview Press; 2007.

29. Zahariadis N. Ambiguity and multiple streams Boulder Westview Press; 2014.

30. Kingdon J. Agendas, alternatives and public policies. 2nd ed. Boston: Little, Brown and Company 1995.

31. Schagler E. A comparison of frameworks, theories anf models of policy processes. In: Sabatier P, editor. Theories of the Policy Process Boulder Westview Press 2007.

32. Brunner S. Understanding policy change: Multiple streams and emissions trading in Germany. Glob Environ Change. 2008;18(3):501-7.

33. Ackrill R, Kay A, Zahariadis N. Ambiguity, multiple streams, and EU policy. J Euro Public Polic. 2013;20(6):871-87.

34. Jones MD, McBeth MK. A Narrative Policy Framework: Clear Enough to Be Wrong? Policy Stud J. 2010;38(2):329-53.

35. Cairney P. Understanding Public Policy: Theories and Issues. Basingstoke, UK: Palgrave MacMillan; 2012.

36. Mettler S, Sorelle M. Policy Feedback Theory In: Sabatier P, Weible CM, editors. Theories of the policy process2014.

37. Heclo H. Modern social policies in Britain and Sweden New Haven Yale University Press; 1974.

38. Dowding K. Model or metaphor? A critical review of the policy network approach. Political Studies. 1995;43(1):137-58.

39. Baumgartner FR, Jones BD, Mortensen PB. Punctuated Equilibrium Theory: explaining stability and change in public policymaking. In: Sabatier P, editor. Theories of the Policy Process. Boulder Westview 2014.

40. Eriksson L. Rational choice theory: potential and limits London: Palgrave Macmillan 2011.

41. Ward H. Rational Choice Theory In: Marsh D, Stoker G, editors. Theory and methods in political science Basingstoke Macmillan 2002.

42. Schneider A, Ingram H, de Leon P. Democratic Policy Design: Social Construction of Target Populations. In: Sabatier P, editor. Theories of the Policy Process. 2nd edition ed. DOI: 10.1111/psj.120542014.

43. Schneider A, Ingram H, DeLeon P. Democratic policy design: social construction of target populations In: Sabatier P, Weible CM, editors. Theories of the policy process. Boulder: Westview Press; 2014.

44. Pierce JJ, Siddiki S, Jones MD, Schumacher K, Pattison A, Peterson H. Social Construction and Policy Design: A Review of Past Applications. Policy Stud J. 2014;42(1):1-29.

45. DeLeon P, Varda DM. Toward a Theory of Collaborative Policy Networks: Identifying Structural Tendencies. Policy Stud J. 2009;37(1):59-74.

46. Bletsas A, Beasley C. Engaging with Carol Bacchi. Adelaide University of Adelaide Press 2012.

47. Bacchi C. Policy, theory, politics: problem representations in drug and gambling policy Adelaide Adelaide University 2006.

48. Bacchi C. Women, policy and politics: The construction of policy problems London Sage Publications 1999
