# Supplementary material for: The application of theories of the policy process to obesity prevention: a systematic review and meta-synthesis
Source: BMC Public Health. 2016 Oct 13;16:1084. doi: 10.1186/s12889-016-3639-z (PMC5064928; doi:10.1186/s12889-016-3639-z)
Supplement: Additional file 2: — Search strategy. (DOCX 18.2 kb) [file 12889_2016_3639_MOESM2_ESM.docx]

**Search Strategy**

**Stage 1- Identification of theories of the policy process**

Policy focused journals search for the terms “policy process theory” OR “theories of the policy process” included:

- Health Policy and Planning
- Health Policy
- Journal of Health Politics and Law
- Journal of Public Health Policy
- Journal of Social Policy
- Policy and Politics
- Policy and Society
- Policy Studies
- Political Sciences
- Public Administration Review
- Social Policy and Administration
- Milbank Quarterly

**Stage 2 – Application of theories of the policy process**

(((Obes* OR overweight OR “body weight” OR “weight loss” OR “body mass index” OR BMI) OR (Food OR diet* OR Nutrition* OR “soft drink” OR “sugar sweetened beverage” OR fat OR sugar OR salt OR “energy dense” OR “fast food” Or “take away”) OR (Physical activity OR leisure OR sedentary OR exercise)) AND (Policy OR “policy process*” OR “policy development” OR “policy adoption” OR “policy enactment” OR “policy decision making” OR regulation OR legislation)) AND (“policy theor*)” OR “Advocacy coalition framework” OR “advocacy coalition theory” OR “Sabatier” OR “multiple streams theory” OR “Multiple streams framework” OR “three streams model” OR “three streams framework” OR “Kingdon” OR “Punctuated equilibrium theory” OR “Baumgartner and Jones” “Institutional Analysis and Development OR IAD” OR “Ostrum” OR “Institutional* theory” OR “garbage can model” OR “actor network theory” OR “theory of collaborative policy networks” OR “policy network theory” OR “Marxism” OR “neo-liberalism” OR “Diffusion of innovation” OR “Narrative policy framework” OR “Policy feedback Theory” OR “Social construction framework” OR “social construction theory” OR “Rational choice theory” OR “Incrementalism” OR “Agenda setting theory” OR “Bacchi’s theory” OR “Bacchi’s approach” OR “What is the problem represented to be” OR WPR OR “health policy analysis triangle”))): Boolean/Phrase search with no limiters applied.

**Other databases included in search were:** AgeLine, AHFS Consumer Medication Information, AMED - The Allied and Complementary Medicine Database, Applied Science & Technology Source, Art Source, Audiobook Collection (EBSCOhost), Avery Index to Architectural Periodicals, Business Source Complete, Communication & Mass Media Complete, Criminal Justice Abstracts with Full Text, eBook Academic Collection (EBSCOhost), EconLit, Education Source, E-Journals, Environment Complete, ERIC, European Views of the Americas: 1493 to 1750, Garden, Landscape & Horticulture Index, Global Health, GreenFILE, Health Business Elite, Health Policy Reference Center, Health Source - Consumer Edition, Health Source: Nursing/Academic Edition, HEED: Health Economic Evaluations Database, Historical Abstracts with Full Text, Humanities Source, Inspec, International Bibliography of Theatre & Dance with Full Text, Jewish Studies Source, Legal Source, LGBT Life with Full Text, Library & Information Science Source, Library, Information Science & Technology Abstracts, MAS Ultra - School Edition, MasterFILE Premier, MLA Directory of Periodicals, MLA International Bibliography, Newspaper Source Plus, Newswires, Philosopher's Index, Political Science Complete, PsycARTICLES, PsycBOOKS, PsycEXTRA, Psychology and Behavioral Sciences Collection, PsycINFO, PsycTESTS, Regional Business News, Religion and Philosophy Collection, Social Work Abstracts, SocINDEX with Full Text, SPORTDiscus with Full Text, The Serials Directory, Urban Studies Abstracts, Web News
