# Supplementary material for: The application of theories of the policy process to obesity prevention: a systematic review and meta-synthesis
Source: BMC Public Health. 2016 Oct 13;16:1084. doi: 10.1186/s12889-016-3639-z (PMC5064928; doi:10.1186/s12889-016-3639-z)
Supplement: Additional file 3: — Critical appraisal rating system. (DOCX 19.3 kb) [file 12889_2016_3639_MOESM3_ESM.docx]

| **Critical appraisal construct reviewed** | **Credibility**  *Do the findings represent the views of participants?*  Evaluation of credibility of each study required an assessment of whether the following was included:   - Use of verbatim quotes - independent analysis of data by more than one researcher and/or employ outside auditor of analysis (member checks) - participants validated findings - peer debriefing - attention to negative cases - persistent observation | **Transferability**  *Were there contextual details provided?*  Evaluation of the transferability of each study required an assessment of the whether the following was included:   - Participant demographics - Thick description of study context | **Dependability**  *Was the process logical, traceable?*  Evaluation of dependability required an assessment of whether there was evidence of:   - Peer review - Triangulation (data, researcher or theory) - Calculation of inter-rater agreements | ***Confirmability***  *Are findings qualitatively confirmable through an analysis of audit trail?*  Evaluation of the confirmability of the study required an assessment of whether the study included details of:   - Effects of the researcher during all steps of the research process (reflexivity) - Provision of information on the researcher background information education, perspective, school of thought that may influence the analysis and reporting of results |
| --- | --- | --- | --- | --- |
| **Evidence required for each scoring level** | H= multiple methods outlined above were employed in combination (e.g. peer debriefing, auditing and participant validation were used)  M= Only one or two methods outlined above were employed.  L= Minimal or no evidence of the use of methods outlined above in the study | H= participants demographics provided and a thick description of the context were both provided (as suggested by Yin (2014);  M= either demographics or thick description missing from study or both of these aspects were incomplete in nature  L= both a description of the participant demographics and thick description are missing from the study | H= There was evidence of the use of two or more methods outlined in the study  M= There was evidence of one of the above methods  L= Minimal or no evidence to suggest the above used in the study | H= Details of researcher perspective are included in the study and strategies to enhance reflexivity are noted  M= One of the two above were documents  L= Minimal or no evidence that the above strategies were used. |
