# Supplementary material for: The application of theories of the policy process to obesity prevention: a systematic review and meta-synthesis
Source: BMC Public Health. 2016 Oct 13;16:1084. doi: 10.1186/s12889-016-3639-z (PMC5064928; doi:10.1186/s12889-016-3639-z)
Supplement: Additional file 4: — Detailed findings by study. (DOCX 50.9 kb) [file 12889_2016_3639_MOESM4_ESM.docx]

| **Author, year** | **Theory used** | **Influences on policy processes** | | | | | | | | |
| --- | --- | --- | --- | --- | --- | --- | --- | --- | --- | --- |
|  |  | **Coalition/ industry group influence** | **Use of evidence** | **Political institutions and political systems** | **Narrative and framing** | **Personal values and beliefs** | **Prevailing Political ideology** | **Timing** | **Leadership of key individuals** | **External socio-political factors**  **Other** |
| Craig et al. 2010 | MST | Differing perspectives by public health advocates: Those who participated in the Task Force and the Arkansas Preventive Nutrition and Physical Activity Summit generated progressive policy alternatives however some others public health professional’s advocated for only incremental change. Without the political leadership demonstrated the status quo may have remained. The lens of the Multiple Streams Framework, the influence of public health professionals is clearly seen in raising awareness and proactively generating policy alternatives. | Childhood obesity indicators were used to garner support for effort to combat the epidemic. However the role of evidence in formulating policy responses was less clear with the policy options developed from taskforce meetings. | Policy change was enabled by expanding upon an existing program that was in place. Feasibility was considered throughout the policy development process as well with one of the major summits that lead to reform only asking leaders who were thought ‘‘most able to initiate and implement change. At the forum they were tasked with devising practical, achievable policy alternatives for education, including school environment. |  | The speaker of the house experienced serious obesity-related personal health problem, which served as a focusing event that brought attention to the seriousness of the issue. The politicians experience with obesity related illness meant that decision makers were willing to hear about new and innovative ways to address the problem, and were prepared to take the political risk.  Timing of the senior democrat experiencing obesity related illness meant that decision makers were willing to hear about new and innovative ways to address the problem, and were prepared to take the political risk. |  | The timing of a Preventive Nutrition and Physical Activity Summit helped to harness advocacy for policy change. | A policymaker was motivated to action by concern about the amount of caffeine and sugar in foods and beverages available to children in school vending machines.  Determined to uncover possible solutions, the respondent led legislative hearings to raise awareness of the issue and generate policy alternatives. |  |
| Dodson et al. 2009 | MST | Lobbying manufacturers of unhealthy foods and beverages is a strong influencer of the introduction and adoption of childhood obesity prevention legislation’ |  | **Feasibility-** “A broad group of individuals working on the actual language of the bill, what need[ed] to be included, rather than someone saying, ‘Oh, this is a great idea. Let me sit down and write a bill about it.’ But the more people you involve, the more buy-in that you get, the stronger the coalition that you have supporting it, certainly the easier it’s going to be to pass”  **Feasibility (cost):**  cost was a primary concern to decision makers. | Citizen understand-ing of the issue, or rather lack of was cited as a barrier in ensuring political feasibility of obesity prevention legislation. (schools and parents were opposed to the legislation due to a lack of understand-ing of the potential to improve health and its limited negative impact on schools or local economic) Therefore strategies to change the narrative of the causes of obesity were suggested | **Personal experience with issue**- Introduction by senior legislators who had a strong personal interest | Despite a “democratic-controlled legislature, the Assembly Health Committee was, for a period of time, the greatest challenge to good policy” (why could be due to political ideology or institutional factors- this was not discussed in the findings) |  |  |  |
| Freunen-berg et al. 2015 | MST and ACF | There was evidence of food-related advocacy coalitions which were demonstrated to influence policy agendas. In addition, officials enlisted numerous food-related activists and community organizations in developing their food plans, helping to bring these individuals and groups into the political arena and perhaps convincing other candidates that food and food inequalities were viable issues. These campaigns also served to create (in Sabatier's term) an emerging food policy advocacy coalition that would play an important role in future policy timing opportunities (i.e. changes of government).”  “The London Food Strategy was in its second implementation phase and the London Food Link and Sustain, the partners involved in its implementation, which were instrumental in raising food related questions.” On the policy agenda. |  | **Feasibility**  In London, the Food Strategy is still in place and there is a renewed focus on its implementation through the London Food Board and its three implementation groups, which had strategies already in place that could be used at a broader scale for policy implementation. | Over time, food policy debates became more prominent in Mayoral politics in both cities. While much of the media attention focused on the ‘headline grabbing’ issues such as Mayor Bloomberg's proposed limitations on the size of soda portions, policy makers, advocates and journalists in both cities now see food policy as a legitimate domain of city politics.”  “The nature of discourse changed over time with food security, food waste and environment-al sustain-ability, the working conditions of food workers and the impact of climate change on food becoming more salient issues for mainstream consideration, although there is still an emphasis on the priority of reducing obesity.”  Election media coverage of food policy increased over time and was associated with changes in decision making. For example, newspapers ran articles on the candidates' positions on outgoing Mayor Bloomberg's proposal to limit the size of sugary beverage containers as a way of reducing caloric intake. Several media outlets covered the Mayoral food policy forum which legitimised food policy as an election issue and also normalized municipal involvement in food policy, a powerful challenge to the prevailing belief that food choice was solely an individual responsibility  There was also evidence of **alignment** of the narrative with other issues particularly sustainability |  |  | The themes of fairness and social justice in the broader political environment) provided food justice advocates with a window of opportunity to make their case. Food justice advocates in New York were in a much better position than at any time in the last decade to organize to pressure city government to make food policy a higher priority issue. In both locations food advocates and sympathetic elected officials used the interval between the two elections to develop and make a case for a variety of policy ideas. The second election provided the opportunity to present these proposals (e.g., free school lunch for all and protecting children from junk food) to the candidates, the media and the public. |  |  |
| Gladwin et al 2008 | MST | In comparison to the organisations who were involved in the successful policy adoption, the organisation promoting the unsuccessful policy was small and lack policy advocacy capacity and the support of decision-makers and policy influencers within the policy decision-making context (education system). | Evidence of physical activity contributing to enhanced learning outcomes was used to strengthen arguments for the policy approach, as well as for rebutting against opposition by teachers (potential implementers of the policy) that it would lead to poorer educational outcomes.  For the policy that was not adopted there was no evidence of effectiveness of the approach. | **Feasibility:** For the policy that was adopted: Limited funding for implementation of the program was identified as an issue but evidence of need and potential effectiveness used to strengthen arguments for the policy resulting in them outweighing feasibility concerns.  No requirement for formal evaluation and leaving many aspects of the implementation up to individual teachers were also important factors to the policy.  The policy that was not adopted was viewed as not being feasible due to the capacity required to implement within schools (needing substantial funding).  In comparison with the unsuccessful policy initiative, the successful policy avoided straining the education system’s capacity by closely aligning with existing policy, and hence was perceived to be implementable in the short term. | Minister for Learning framed the policy issue as the need for increased physical activity in schools. | A physician with personal beliefs about the benefits of physical activity took office as the Minister for Learning. This enabled the preferred policy option to be adopted. |  |  |  |  |
| Gomez 2015 | Institutional theory | In Brazil (where obesity prevention policy has progressed), the ministry of health created an important systems structure: the National Policy of Nutrition). This was a result of a congress and substantial consultation with experts, civic and private industries. This was integral for implementing policy actions developed, as well as creating networks and partnerships for ongoing work. |  | Access to decision-making points was identified as a key difference that contributed to the variance in policy adoption between the USA and Brazil, suggesting that “the implementation of public health policy faces fewer hurdles in Brazil.”  The decentralization of public health policy responsibility, and the fact that Brazil’s ministry of health has a much longer tradition of intervening and coordinating with the states to implement public health legislation was seen as an enabler (this was linked to considerations of feasibility). |  | Personal interest: The author suggests that both Bush and Obama administrations’ in obesity motivated them to initiate federal campaigns and legislation. | The fact that Brazil’s Public Health Agencies are more successful at securing congressional funding and political support for policy implementation and the US was identified as a key barrier for obesity prevention policy implementation [Also relevant to institutional factors].  The authors explain “..that this is in large part the result of pre-existing normative commitments to the provision of universal healthcare as a human right, therefore the ongoing financing of public health policies. In contrast, the DHHS in the US has at times in the past faced considerable difficulty obtaining financial support for new policies [60]; the absence of a shared normative commitment to healthcare as a human right, and conflicting partisan views over the government’s role in public health appear to have been the main culprits.”  The historical context of obesity prevention policy in the US saw the absence of obesity’s effects on the US national security and to the economy resulting in minimal political engagement. |  | In Brazil political leaders used media to increase family awareness and interest in obesity prevention. The USA in contrast did not. |  |
| Houlihan et al. 2006 | MST and ACF | There was little evidence to support the view that the increased salience and status of school sport and PE was the result of sustained policy advocacy from dominant coalitions or actors/ organisations. Instead the study suggests there was a distinct lack of coalition behaviour centering on consensus of values/ beliefs that may have helped policy change earlier. Influential single organisations who presented feasibility policy solutions were more influential in policy decision making.  E.g. “From what I can gather there are quite strong tensions between [coalition 1] and [coalition 2] and if that is the case then that will weaken their argument’”  It was identified the lack of power of lobby group was due to “awful leadership at the time”. | The authors emphasized that those influencing policy were able to align their evidence against the objectives of the government [not their own].  Three types of evidence were important in policy change. 1) Evidence relating to the magnitude of the problem provided separately by interest groups; 2) evidence in regard to other problems (provided from other departments) for which the policy could be tied to as a solution, 3) the third was the evidence of effectiveness of the policy against each of the problems. | Authors suggest that the absence of an institutional focus for the policy issue (school sport and PE) within the central government department may help to explain the persistence of a lack of core policy values and beliefs. This was compounded by institutional weakness of the Department within which the policy portfolio resided.  Feasibility was important with a policy adaptation considered to be the result of there being stakeholders’ capacity and willingness to deliver what the government department wanted delivered.  Another key factor clearly linked to policy change was the change in location within government in terms of the responsibility from a department limited in power to the Prime Minister’s Delivery Unit. | An influential institution was able to able to clearly convey messages and values conveyed by the organization that the policy would help government achieve policy goals that extend beyond a narrow focus of the policy (school sport and PE).  The policy entrepreneur Identified that, in order to achieve the policy goal, the policy debate had to be expressed in a different language.  In the ensuing debates in the media and among politicians much was made of allegations that competitive team games in schools were being undermined by ‘wet liberal’ PE teachers. What followed was described by Evans (1990) as ‘something of a moral panic about the teaching of Physical Education’ (p. 155).  **Media (not a strong influence)**  The fear of exclusion from the National Curriculum, the media-generated concern with the decline in competitive school sport or the introduction of National Lottery funding are all possible exogenous factors, but none is a particularly compelling explanation |  | There was little evidence of much coalition ‘members’ shared beliefs over core policy matters’ which provide ‘the ‘‘glue’’ that holds an advocacy coalition together’ | Policy entrepreneurship was evident “at the right time” to ultimately enable policy change. Evidence also suggested that this was largely about networks and connections [and] the ability to capitalise on them. | In the absence of influential coalitions, arose individual- policy entrepreneurs that lead the policy change. The roles of individuals were critical in shaping the policy. Interest in the issue from key politicians was a key aspect of this. The Authors contend that policy entrepreneurship requires a supportive context. | The Olympics as an event helped to increase awareness and to enable the issue to climb higher on the policy agenda. |
| Khayesi et al. 2011 | MST | There were competing coalitions evident. However opposing coalitions were able to be overcome differences through practical demonstration the there would not be negative financial impact of the policy (not feasible for many obesity prevention policies) |  | Feasibility was important with “sign-off” occurring only when the minister was convinced that the plan was viable. This did require the creation of an institution responsible for the delivery.  The integrated transport and planning structure of the government also assisted policy progress for active transport. |  | Related to the influence of key individuals although the values and beliefs were not explored in the analysis this area is therefore assumed. | Changes in the power of departments responsible for the policy were also influential in shaping policy decision-making. | Timing was mentioned, in that political appointments created the opportunity for progress but this was more in respect to influential leaders shaping policy rather than serendipitous alignment of policy, problems, and politics to enable policy change. | Significant influence of individuals: For many of the key policy decisions, key individuals were critical in policy outcomes (most often the individuals were Ministers with decision making authority). “E.g. the policy change came about because the Secretary of State [for the DCMS] at the time was trying to respond to the Prime Minister’s desire to do something about school sport.” |  |
| McBeth et al. 2013 | NPF |  |  |  | Individual framing of issues compared to societal means the perceived as popularity of regulatory policy changes is limited. |  |  |  |  |  |
| Milton and Grix 2015 | MST | Lobbying efforts were undertaken by policy entrepreneurs and groups who were proponents of particular policy action (e.g Walk England) | The lobbying efforts of these policy entrepreneurs was facilitated growing research evidence on the health benefits of walking and prevalence data on rising levels of inactivity. However in terms of what policy instrument provided the best approach (according to the evidence), it was concluded that politics and professional judgement, rather than research evidence alone was the key influence. | Technical feasibility:  The adopted policy (Walking for Health) had a proven track record of feasibility. | The Issue was defined or ‘packaged’ in three primary ways: as a health issue; a transport issue; and as an environment issue; and this has impacted on how responsibility for walking promotion has been dealt with by the government. This “general-isation of interests” was suggested by the authors as a strategy of policy entrepreneurs to make issue of relevance to a broad audience (range of government departments, in terms of their objectives) to increase the issue salience with decision makers. An additional barrier to walking promotion, which was expressed by representatives from both DH and DfT, is the perception that walking is such a simple behaviour that the general public will not view walking promotion as sufficiently complex or necessitating high level expertise, to warrant political attention, and thus this will not be considered an appropriate use of scarce Government resource. |  | Political ideology was found to be integral given the historical context whereby health promotion was not seen to legitimately fall within the government’s remit. The authors suggested that there was “.a long history of policy in England which emphasises the importance of individuals taking responsibility for their own health behaviours. One of the greatest changes to policymakers was not to convince decision makers of the evidence of the issue but to convince policymakers that the policy (walking promotion) legitimately falls within the government’s remit. | The timing of the Olympics meant that the government were keen to act, and had to identify a policy solution. The policy entrepreneurs (individuals and groups) were able to harness the opportunity to sell their solutions to decision makers (as technically and politically feasible, and affordable. | Advocates for walking promotion were identified as “instrumental in bringing the issue to the attention of government and for encouraging political action…. “It's a fact of life that certain people will like other people and listen to what they say. And it happens more than you could ever believe in terms of someone having the ear of a Minister”. | The Olympics meant extraordinary funding was allocated to meet a new government goal in line with the London Olympics.  There was a Treasury review of the public spending commitments made by the previous government which examined billions of spending that was approved during the previous government’s final few months in office. The aim of the review was to assess whether these commitments were affordable, whether they would deliver value for money, and whether they were considered a priority for the new government. In total 12 projects were cancelled because they were deemed unaffordable and not a government priority, one of which was Walk England’s Walk4Life Miles project |
| Mosier et al 2013 | MST | Business groups opposed the policy and aligned themselves with the opposition political party. The confectionary industry was seen as separate from the soft drink industry in the Colorado experience. Lobbyists representing the beverage industry, grocery stores were more present in the policy process in Kansas and used arguments that the policy would be financially detrimental to companies, which in turn would negatively impact on the community through redundancies. Whereas lobbying by the soft drink industry was absent in Colorado. | Colorado was able to get the policy adopted compared to Kansas DESPITE having one of the lowest obesity prevention prevalence’s in the USA at the time of policy consideration.  Evidence of an issue (in this instance this was a budget deficit) was not sufficiently created in the public sphere in Kansas.  Aligning the policy as a response for budget deficit rather than for a public health response removes the risk for arguments being made against its effectiveness for improving obesity prevalence (for which there is limited evidence of effectiveness available) | **Feasibility-**  Feasibility of policy options influenced policy both case study policy processes. Public discussions (criticisms) of the feasibility of implement-ation was associated with one of the policies not being able to be adopted. This was largely due to a slightly different policy instrumentation (sales tax compared to a reversal of a tax exemption that included other products apart from sugar sweetened beverages). The sales tax in Kansas was argued to be too difficult to implement with classifications of foods being too onerous for food outlets. Whereas proponents of the Colorado tax were suggested to be effective at demonstrating feasibility. This was linked to an increased lead in time and consultations, which led to proponents being able to seek legal counsel to ensure compliance with legislation and assessing ease of administration. The cost to implementation was also minimal.  Political system feasibility was initially an issue with opponents suggesting that the state constitution, with it encroaching on the “Taxpayer’s Bill of Rights (TABOR), a revenue- and expenditure-limitation measure.”  Policy actors suggested that it was unclear which ideas were viable solutions. | In both cases the debate was split down political parties lines. In the case where the policy was adopted, the political leader was very careful in framing the policy as being for budget purposes only, whilst opponents tried to cite that it was due to interest in public health to shift the narrative to as impost on individual freedom, to garner public opposition to the policy.  “The media and interest groups tried to cite the Governor’s interest in public health as evidence of the governor’s motive to socially engineer the state to be healthier.”  Multiple media sources declared that the policy highly unpopular with citizens and businesses in Kansas because of the potential economic impact and unnecessary intervention by government in individual consumption choices. |  | The political atmosphere surrounding policy was divided according to political ideology. There was policy adoption in one of the two states (Colorado) despite this being a dominated by anti-government and anti-tax sentiments. However this state did not have to contend with a split majority party or other political difficulties that were present for Kansas (the policy ended up being relayed to the Senate Assessment and Taxation committee, which was controlled by conservative Republicans who opposed the legislation). |  | Swift and persuasive leadership enabled policy action, through discussing policy alternative early with the policy community and normalising their preferred policy solution. | **Exogenous factors** - Budget deficit lead to the need to generate revenue, which led to the consideration of a number of taxation policies. Sugar sweetened beverages were one such policy. |
| Olstad et al 2015 |  | Public health coalition groups were important for a number of the states in advocating for policy change (for physical activity to be included within school curriculum/quality standards). | There were a number of high profile reports that provided evidence of inadequate PA to childhood obesity and poor health outcomes, which ultimately lead to “policy windows” to open to enable policy change. | Feasibility of policy options was important for policy progress. For example in Alberta, the policy instrument (school based physical activity) was argued by proponents as a viable and effective means to address the problem (via increasing PA). |  |  |  |  | The Minister for learning presented the policy as a viable solution to the problems of inactivity and obesity among children. |  |
| Quinn et al 2015 | MST | Coalitions were not described specifically, however, industry and public health groups were noted as influencing policy with the beverage industry meeting with the minister’s office to object the policy change. | Public health surveillance indicated that obesity which translated to it being a high priority. | **Feasibility:** The study found that the vending machine and nutrition guidelines provided a feasible alternative to regulations that ad- dressed the problem of unhealthy food away from home. | Board members framed the issue about personal choice rather than implementing restrictions or bans. |  | The region had a history of using policy to address public health issues, including trans-fat in restaurant food, menu labeling, healthy community planning, and tobacco use. | A “window of opportunity which we anticipate will eventually close because the board’s attention will change to something else.” | Several board members had a history of being active in local food system and policy development. |  |
| Phillpots 2013 | ACF | The involvement of a number of groups or powerful elite was clearly evident in the policy development and adoption. A key policy entrepreneur was able to construct a coalition that was successful in lobbying the relevant government departments to adopt the preferred policy options.  The bringing together disparate and often competitive assortment of organizations, to work together in order to achieve a broad set of mutually shared outcomes was beneficial for the policy adoption.  The coalition pressuring decision makers to ultimately influencing the policy decision. This coalition was able to subdue other policy interest groups, although there was no evidence of any coalition actively opposed to the policy entrepreneurs ideas. | Occurrence of policy learning was evidence. | **Feasibility** was a key influence on policy with a group that had demonstrated ability to implement their preferred policy option. | A policy entrepreneur was active in providing a clear vision for the policy issue to politicians | The key policy entrepreneur was able to align the value systems of senior politicians with her vision for the policy. Therefore there was no empirical evidence of change in beliefs that is described by Sabatier’s ACF. | A shift in the government of the day, resulted in an ideological shift towards more market based policy instruments (whereby schools would be incentivized to perform in respect to the policy issue compared to being enforced to meet certain standards) | The Olympics was a key external factor that meant that an policy opening for policy change occurred. This was in addition to a new government and a system structure that enabled policy action.  The global financial crisis also influenced the policy with retracting government budgets meaning the policy funding was retracted. | Key individuals were important with the prime minister a supporter and advocate of the policy. | The proposed and endorsed policy options built on growing political and popular interest in physical activity following the London Olympic Games |
| Reid and Thornburn 2011 | MST | Certain individuals/ groups had greater access and influence in the policy process: The weakness of the sports lobby at the organisational level and lack of embeddedness within government compared with established lobby groups was a barrier. With one civil servant arguing ‘there are too many individuals and small groups with their own agendas and ministers easily flick them away’. |  | Feasibility within the political cycle time period was a factor considered, to ultimately shape the policy outcome.  Policy capacity (ability to recognize an opportunity to influence policy) was another identified as another critical aspect for preferred policy instruments to be adopted.  Administrative turnover, which was linked to risk aversion impeded policy innovation. | A lack of a consistent voice regarding the policy issue was identified as a reason for policy resistance. |  | The policy instrument was said to lack value acceptability with decision-makers, which limited policy progress.  **Personal experience-** The personal experience with the policy issue (in this case PE) influenced the decision-making processes of politicians and senior officials involved in the policy formulation. | The role of passionate leadership which focused on ideology was evident. Advisors then tried to tie the evidence to these ideas about the policy options. |  |  |
| Thow et al 2014 | Health Policy Analysis Triangle | The Ghana Health Service and Ministry of Health played a key role in  identifying the need for the standards. Interviewees also reported that the standards were the result of collaboration between the Ministry of Trade issued the directive for their development (GSA is an implementing agency of the Ministry of Trade).  The Ministry of Trade reported that membership of the GATT/WTO influenced the need for the policy and the formulation of the policy. | Evidence of the NCDs, and linking this to fat intake was important for getting the policy onto the agenda.  Influential groups advocated for an evidence based standard for reducing fatty meat imports. | The successful initiation, de- velopment and implementation of the policy required collaboration at every stage between Ministries of trade and health.  There were also technical committees that developed the standard, which included representatives from Ghana Standards Authority, Food and Drug Authority, the Ministries of Health, Trade and Agriculture, the Council for Scientific and Industrial Research, universities, and other agricultural research institutes. | There was a public narrative around the issue with public concern about high-fat diets (particularly saturated fat) in relation to rising rates of NCDs. This seemed to be part of a broader perception that imported meat was of low quality |  |  |  |  | There were reports that the USA had suggested removal of the standard, and this was attributed to the World Bank (this finding was not supported by document analysis however). Ghana’s membership in the WTO did have an effect in that membership in informing the policy formulation. |
| Ulmer et al 2012 |  | Broad-based stakeholder buy-in was a main facilitator to successful legislation enactment. |  | Administrative and political delays, as a result of staff turnover were a barrier.  Three institutional barriers were present; these were in relation to staff turnover, a lack of a consistent policy champion, and bureaucratic risk aversion.  Whilst key champions helped the policy enactment, it was the restructuring of this very bureaucracy that removed critical support and delayed progress.  **Feasibility**-The reassurance to stakeholders that such an initiative could be successful in this context, through a successful implementation in another state also enabled the enactment. Findings also suggest that information and analysis was important. |  |  |  |  | Political champions were integral, “both on the council and in city government, who endorsed the measure from the beginning and pushed hard to make it happen”. | A fınal impediment to policy progress initiative was the existence of many competing priorities in New Orleans following hurricane Katrina.  There was a window of opportunity that opened natural disaster, which actually resulted in significant civic engagement by citizens. |
| Yeatman, 2003 | Agenda Setting theory |  | In all policy examples, in-depth studies of local food and nutrition issues had been undertaken. A range of local data had been presented to the local governments, including comparative health studies, dietary intake studies and food access reports. These data highlighted the problems and issues experienced by local residents, such as limited access to food retail outlets, poor quality fruit and vegetables, high food prices in economically depressed areas and significant populations with high nutritional needs.  The data were presented in ways that identified how local government action could impact on the situations reported. For example, planning requirements could include requirements for parenting facilities in public buildings to support breast-feeding, and economic development plans changed to include support for a second super-market in an area to promote competition and hence lower food prices for residents. | Policy initiatives did not result in acceptance of formal policies by the local governments, due in part to significant organizational changes that occurred at those sites. For example, one local government was amalgamated with another larger local government and its food policy agenda was abandoned.  Committees that cut across the traditional hierarchical structures within the local governments were advantageous to policy progress. Therefore action by individuals to influence institutional arrangements (cross-department policy committees) was hypothesized an effective mechanism to bring about policy change  Traditionally local governments have a clear line-management arrangement, based around their legislative responsibilities. Such arrangements are not supportive of developing broadly based health policy. |  |  |  |  | In two case studies, the health sections had been reorganized to free some senior staff from much of their direct service delivery and enable them to take on different roles. This had resulted in specific staff members being allocated tasks, such as executive support for the food and nutrition policy committees and activities such as research and policy develop-ment. | Given the location of the study was within local government, an external factor was the funding support from the Commonwealth department of health as part of the National Food and Nutrition Policy implementation strategy |
